# Large-scale survey to estimate the prevalence of disorders for 192 Kennel Club registered breeds

**DOI:** 10.1186/s40575-017-0047-3

**Published:** 2017-09-19

**Authors:** B. M. Wiles, A. M. Llewellyn-Zaidi, K.M. Evans, D. G. O’Neill, T. W. Lewis

**Affiliations:** 1 The Kennel Club, Clarges Street, London, W1J 8AB England, UK; 2 International Partnership for Dogs, 504547 Grey Rd 1, Georgia Bluffs, ON England, UK; 3 School of Veterinary Medicine and Science, The University of Nottingham, Sutton Bonington Campus, Sutton Bonington, Leicestershire, LE12 5RD England, UK; 4 Veterinary Epidemiology, Economics and Public Health, Royal Veterinary College, London, NW1 0TU UK

**Keywords:** Prevalence, Morbidity, Dogs, Survey, Breeds, Pedigree

## Abstract

**Background:**

Pedigree or purebred dogs are often stated to have high prevalence of disorders which are commonly assumed to be a consequence of inbreeding and selection for exaggerated features. However, few studies empirically report and rank the prevalence of disorders across breeds although such data are of critical importance in the prioritisation of multiple health concerns, and to provide a baseline against which to explore changes over time. This paper reports an owner survey that gathered disorder information on Kennel Club registered pedigree dogs, regardless of whether these disorders received veterinary care. This study aimed to determine the prevalence of disorders among pedigree dogs overall and, where possible, determine any variation among breeds.

**Results:**

This study included morbidity data on 43,005 live dogs registered with the Kennel Club. Just under two thirds of live dogs had no reported diseases/conditions. The most prevalent diseases/conditions overall were lipoma (4.3%; 95% confidence interval 4.13-4.52%), skin (cutaneous) cyst (3.1%; 2.94-3.27%) and hypersensitivity (allergic) skin disorder (2.7%; 2.52-2.82%). For the most common disorders in the most represented breeds, 90 significant differences between the within breed prevalence and the overall prevalence are reported.

**Conclusion:**

The results from this study have added vital epidemiological data on disorders in UK dogs. It is anticipated that these results will contribute to the forthcoming Breed Health & Conservation Plans, a Kennel Club initiative aiming to assist in the identification and prioritisation of breeding selection objectives for health and provide advice to breeders/owners regarding steps that may be taken to minimise the risk of the disease/disorders. Future breed-specific studies are recommended to report more precise prevalence estimates within more breeds.

**Electronic supplementary material:**

The online version of this article (doi:10.1186/s40575-017-0047-3) contains supplementary material, which is available to authorized users.

## Plain English Summary

It is often repeated that pedigree or purebred dogs have an unacceptably high occurrence of disorders due to inbreeding and selection for exaggerated features. However, there are few single studies that report the occurrence of all disorders across multiple breeds; instead most focus on specific breeds or specific conditions. Information on the frequency of various conditions is important in prioritizing health concerns in dogs. Therefore, a survey of dog owners was undertaken to gather health information on Kennel Club registered pedigree dogs, regardless of whether or not these disorders were diagnosed at a veterinary practice. The purpose of this study was to identify the most common conditions affecting the current Kennel Club registered pedigree dog population.

From a total of 43,005 live Kennel Club registered dogs, just under two thirds of live dogs had no reported disorders. The most commonly reported diseases/conditions for all live dogs across all breeds were lipoma (fatty masses) (4.3%), skin cysts (3.1%) and allergic skin disorder (2.7%). Differences between the within breed prevalence and overall prevalence across breeds of some disorders were found. The results from this study will substantially contribute to the current understanding of disorder occurrence in UK dogs. It is anticipated that these results will assist the forthcoming Kennel Club Breed Health & Conservation Plans that will identify and prioritise the most pressing welfare concerns and provide advice to breeders/owners on steps that may be taken to minimise the risk of common conditions in particular breeds. Future breed-specific studies are recommended to gather more precise health information across more individual breeds.

## Background

The domestic dog (*Canis familiaris*) is the most morphologically diverse animal species, and consequently there is likely to be considerable variation in morbidity both within and between breeds [1]. Pedigree or purebred dogs are often stated to have a high incidence of disorders, many of which are popularly ascribed to inbreeding resulting from closed studbooks and selection for over-exaggeration [2–4]. Although many studies have examined the inheritance of conditions which are widely accepted as posing a particular welfare problem in some breeds, there are relatively few studies which empirically determine the prevalence of disorders in general within and across breeds [2, 5–7]. However, those studies that have been published do identify differential prevalence of some disease/conditions across breeds, suggesting some associations between genetic variation and morbidity [2, 5]. Accurate and generalisable prevalence data are critically important to weight and prioritise multiple health concerns and to monitor changes over time [3]. In the last 10 years, three reports into pedigree dog breeding have all identified a crucial lack of accurate prevalence information for disease in UK dogs that have constrained efforts to improve breed health [3, 4, 8].

In recent years, an increasing number of studies on the morbidity of dog breeds have been based on data gathered from primary-care veterinary practices [6, 9–19]. Primary-care practice data have the advantage of providing accurate information on the occurrence of disease in the general dog population (at least those registered with a veterinary practice), with a broad range of conditions being recorded at the point of care by the veterinarian [20]. These conditions will have varying welfare impact depending on their prevalence, duration and severity [21]. Although the classic conditions commonly associated with specific dog breeds, such as epilepsy and syringomyelia, may often have high welfare impact on the individual dogs that are affected because they are severe or life-threatening, the reality may be that their impact at an overall species or even breed level may be greatly diminished due to low prevalence or duration values [21]. Conversely, disorders that are very frequent and may impair large proportions of dog’s lives such as otitis externa, skin hypersensitivity and anal sac impaction are commonly ignored during health prioritisation and research, and yet these apparently ‘minor’ disorders may be considered to be serious issues by owners and may have huge welfare implications for the affected individual as well as for breeds and dogs overall [6]. Gathering evidence on the occurrence of common disorders in the wider population helps to identify these high prevalence ‘minor’ disorders that can contribute substantially to the welfare burden of individual animals or breeds, as well as adding to the financial and emotional cost to owners. While the quality of primary-care data is generally very good, O’Neill et al. [22] note that some misclassification and technical complexities related to the management and analysis of large primary care practice datasets have been reported. Geographical bias and owner socio-economic status may also affect the results. Furthermore, 23% of UK dogs have been reported as unregistered with a veterinary practice and there may be some common disorders which are not often subject to veterinary investigations [23].

Referral-based veterinary clinical studies are valuable in gathering data on specific and usually severe disorders that are more likely to be referred; however, the underlying referral population may be biased towards insured, ill or younger dogs and therefore not be representative of the wider population and thus poorly generalisable for prevalence data [24]. Pet insurance data may also have generalisability limitations, based on owner demographics, with older animals often being uninsured and financial excesses for claims limiting the spectrum of disorders reported [24, 25].

Useful health information on pet animals can also be collected directly from owners to provide data without requiring the intermediary of a veterinary surgery, referral clinic or insurance company. In 2004, the Kennel Club/British Small Animal Veterinary Association (BSAVA) Committee worked with the Epidemiology Unit at the Animal Health Trust to carry out a nationwide survey directly of UK purebred dog owners to identify important health conditions in UK dog breeds. Longitudinal studies, such as Dogslife, a web-based study of Labrador Retrievers, and The Bristol Cats Study [26, 27], have been used to gather data over the lifetime of a cohort of animals but are limited by relatively small sample sizes of animals included. Owner survey data benefit from the inclusion of data on animals which are not necessarily insured or registered with a veterinary surgery and do not need to have experienced any episode of a disorder. Identification and inclusion of dogs with no reported diseases/conditions contributes to more accurate estimates of disorder prevalence in the wider population. Thus, owner surveys have the potential to add to the current knowledge on the common disorders and conditions causing morbidity in UK breeds. With very few breed-wide owner surveys reported and increasing clinical research already being conducted through practice-based surveillance [22, 28], an owner survey methodology was selected for this study. Nevertheless, owner surveys also suffer from many well-recognised shortcomings including as variable response rate, temporality, difficulties in validation and misclassification whereby owners may not recognise a condition as being a true disorder [22].

Consideration of, and comparison between, data reported from multiple sources is likely to offer the most accurate representation of the health of general dog population whilst mitigating the various limitations of each resource. The resultant increase in knowledge on the causes of morbidity in dogs will aid identification of those conditions with the greatest impact on welfare and so assist in the development of more effective strategies to alleviate their welfare impairment.

This study aimed to determine the prevalence of the most common disorders and conditions, as judged by owners, affecting the current Kennel Club registered pedigree dog population and, where possible, identify variations between breeds.

## Methods

### Owner contact and survey distribution

A cross-sectional study was carried out to collect health information on all pedigree dogs currently registered with the Kennel Club on 1st November 2014 and the morbidity data reported here relate to dogs that were alive at the time of the study. The survey was operational online from 8th November 2014 until 31st December 2014. Between 10th November 2014 and 2nd December 2014, 546,836 invitations were emailed to owners of Kennel Club registered dogs with a recorded date of birth in the previous 15 years (from 01/01/1999 to 01/07/2014). Invitations to participate were pre-populated with the Kennel Club registration details of the dog. The survey was also promoted on social media to The Kennel Club followers (on Twitter & Facebook) (between 10th November and 31st December), on the Kennel Club website, in the dog press (Dog World & Our Dogs) and within breeds by personal communication from Breed Health Coordinators. Consequently, some data were gathered on dogs born outside the 15-year threshold. To participate, owners had to access the study online and enter using their dog KC registration name and number.

A prize draw offering three vouchers valued at £200, £100, and £50 for an online retailer was used as an additional incentive to complete the survey. Reminders were emailed to all unopened email addresses and in the dog press at the beginning of December 2014.

Each owner could complete only one survey but could provide information on up to five individual living dogs. Owners that owned more than five live dogs were advised to supply information on the oldest dog, second oldest dog, the median aged dog, second youngest dog and youngest dog.

The survey was applied online using a web-based survey tool (Survey Monkey [29]). An initial draft survey was developed by a team from the Kennel Club, University of Nottingham and Royal Veterinary College. This draft was piloted on 93 people including Kennel Club staff, Breed Health Coordinators and staff at the Centre for Evidence-based Veterinary Medicine (CEVM) study group at the University of Nottingham to test and validate the range of responses and functionality. The pilot helped to refine the format, question types and answer options for the final released survey questionnaire. Sample size calculations estimated that 7299 dogs would be needed to represent a disorder with 5.0% expected prevalence to a precision of 0.5% at a 95% confidence level [30].

### Questionnaire

The questionnaire included 34 individual questions grouped into five sections (Additional file 1). Information was collected on demographics, health, breeding status and behaviour for each of up to five live dogs currently owned and nominated by the respondent.

Section A pertained to general and administrative information on each individual owner and dog (such as Kennel Club registration number, owner email address, date of completion).

Section B gathered information on all disorders and conditions of 16 body systems/condition categories that the dog had been affected by: autoimmune, digestive, oral/dental, heart, respiratory, eyes, skin/coat, ears, bones/joint/muscle, nervous, reproductive, liver, urinary, blood, endocrine, cancers/growths. The order of questions followed a general pattern as shown here for the oral/dental body system:

1. Have any of the dogs included in this survey ever suffered from a serious or persistent oral or dental condition(s)?
2. if ‘yes’ to 1), denote the identity of the dog affected (where the survey was being completed for multiple dogs), and then for each dog affected,
3. provide the age at which the dog was first affected,
4. whether this was diagnosed by a vet,
5. select the specific condition from a predefined shortlist (e.g. oral lump, oral tumour, gingivitis, elongated soft palate, … etc. – for complete list see Additional file 2). The shortlist terminology was developed from the VeNom coding system [31].

Respondents could also add further conditions to those listed in the shortlist provided by selecting the ‘Other condition’ option and specifying the condition via free text as well as the age first affected and whether diagnosed by a vet.

Owners could also specify diseases/conditions outside the 16 available body system/condition categories listed by selecting an ‘Other body system’ option categorised and specifying the condition via free text as well as the age first affected and whether diagnosed by a vet.

Section C pertained to any of the nominated live dogs that were breeding females and included ten questions on health testing, the numbers of litters, puppies born and whether there were any birth defects or congenital conditions affecting the puppies, as well as awareness of Mate Select (the Kennel Club’s online health resource) [32].

Section D pertained to any of the nominated live dogs that were breeding males and contained four questions on health testing and awareness of Mate Select.

Section E contained questions on desirable and undesirable behaviour displayed by dogs owned at the time of the survey.

Owners were asked to be as specific as possible when describing disorders and to use the diagnostic term used by their veterinary surgeon whenever possible. It was also suggested that owners consider contacting their veterinary surgeon to ask for help if they had difficulty recalling precise diagnostic terms. Veterinary surgeons in the UK were informed of the survey via a letter to The Veterinary Record at the start of the study [33].

### Data processing

The online survey closed at 11:59 pm on 31st December 2014 and the data were exported from SurveyMonkey to a spreadsheet in Microsoft Excel CSV format to be cleaned and verified against the Kennel Club database in Microsoft Access [34, 35]. Data on individual dogs were anonymised prior to analysis.

Additional disease/condition terms entered as free text were manually cleaned and categorised where possible via cross-referencing to the lists of conditions developed by one of the authors [BW] prior to the study, built on the VeNom coding system [31]. Novel terms that could not be assigned as a pre-specified condition were added as an additional condition to the total list. Thus as data processing progressed, the list of conditions was iteratively refined and extended as necessary by one of the authors [BW].

### Statistical analysis

Data analysis was carried out in R (an online open-access language and environment for statistical computing and graphics) [36] and Matlab.

Prevalence estimates were calculated by dividing the number of reported cases of a disease/condition in a specified cohort by the number of unique live dogs in the same cohort. The Wilson approximation method was used to calculate 95% confidence interval [37]:

$$ \frac{\left(2 np+{z}^2\pm z\ \sqrt{\left({z}^2+4 npq\right)}\right)}{2\ \left(n+{z}^2\right)} $$

where *n* is the number of responses, *p* is the reported prevalence of the condition in questions, *q* is the prevalence of unaffected dogs (*1-p*), and *z* is the 1-α/2 point of the standard Normal distribution (1.96 for 95% confidence interval).

Prevalence estimates are reported for ‘common disorders’ with ≥50 reported incidents overall (*n* = 101). These estimates are reported as ‘overall prevalence’ based on all dogs included in the survey and also as ‘within breed prevalence’ for each individual ‘common breed’ with responses returned for ≥200 unique dogs (*n* = 41).

For each of the 101 common disorders, significant differences between the within breed prevalence for each breed (*n* = 41) and the overall prevalence estimates were checked using the chi-squared test with Holm adjusted *P*-values to account for multiple testing [37].

The median age at the time of the survey for each of the 41 common breeds and approximate 95% confidence intervals for the median age were calculated in R as:

$$ \frac{1.58\kern0.5em \times IQR}{\sqrt{n}} $$

where *IQR* is the inter quartile range and *n* is the number of responses [38, 39].

## Results

### General results

The survey collected responses representing 43,005 unique live dogs, across 187 breeds (from a total of 215 breeds recognised by the Kennel Club^1^). Of the dogs overall where information was available, 50.88% were male (*n* = 21,882) and 53.53% were neutered (*n* = 23,021). Neuter status was unknown in 1800 animals (4.19%, 920 male, 880 female). The median age of dogs at the time of survey was 4.47 years (interquartile range [IQR] 2.28 to 7.19 years, range 68 days to 28.9 years).

The count of unique live dogs per represented breed ranged from 1 (Canadian Eskimo Dog) to 6938 (Labrador Retriever), with nine breeds yielding data on over 1000 unique live dogs (Labrador Retriever, Cocker Spaniel, English Springer Spaniel, Golden Retriever, Border Terrier, German Shepherd Dog, Cavalier King Charles Spaniel, Miniature Schnauzer, and Border Collie; Table 1).

**Table 1:** Number of unique individual dogs for which responses were received and approximated response rate (from total number registered from 2004–13 inclusive) for all breeds

	#Survey dogs	% Of all survey dogs
Affenpinscher	82	0.19%
Afghan Hound	57	0.13%
Airedale Terrier	186	0.43%
Akita	75	0.17%
Alaskan Malamute	191	0.44%
American Cocker Spaniel	40	0.09%
American Water Spaniel	0	0.00%
Anatolian Shepherd Dog	0	0.00%
Australian Cattle Dog	28	0.07%
Australian Shepherd	71	0.17%
Australian Silky Terrier	0	0.00%
Australian Terrier	12	0.03%
Azawakh	0	0.00%
Basenji	17	0.04%
Basset Bleu de Gascogne	0	0.00%
Basset Fauve De Bretagne	49	0.11%
Basset Griffon Vendeen (Grand)	32	0.07%
Basset Griffon Vendeen (Petit)	48	0.11%
Basset Hound	120	0.28%
Bavarian Mountain Hound	0	0.00%
Beagle	504	1.17%
Bearded Collie	226	0.53%
Beauceron	6	0.01%
Bedlington Terrier	126	0.29%
Belgian Shepherd Dog (Groenendael)	56	0.13%
Belgian Shepherd Dog (Laekenois)	5	0.01%
Belgian Shepherd Dog (Malinois)	27	0.06%
Belgian Shepherd Dog (Tervueren)	88	0.20%
Bergamasco	0	0.00%
Bernese Mountain Dog	190	0.44%
Bichon Frise	263	0.61%
Bloodhound	24	0.06%
Bolognese	25	0.06%
Border Collie	1005	2.34%
Border Terrier	1689	3.93%
Borzoi	65	0.15%
Boston Terrier	130	0.30%
Bouvier Des Flandres	28	0.07%
Boxer	724	1.68%
Bracco Italiano	18	0.04%
Briard	54	0.13%
Brittany	57	0.13%
Bull Terrier	213	0.50%
Bull Terrier (Miniature)	62	0.14%
Bulldog	370	0.86%
Bullmastiff	105	0.24%
Cairn Terrier	299	0.70%
Canaan Dog	6	0.01%
Canadian Eskimo Dog	1	0.00%
Catalan Sheepdog	10	0.02%
Cavalier King Charles Spaniel	1244	2.89%
Cesky Terrier	22	0.05%
Chesapeake Bay Retriever	54	0.13%
Chihuahua (Long Coat)	121	0.28%
Chihuahua (Smooth Coat)	128	0.30%
Chinese Crested	46	0.11%
Chow Chow	79	0.18%
Cirneco Dell’Etna	0	0.00%
Clumber Spaniel	72	0.17%
Cocker Spaniel	3723	8.66%
Collie (Rough)	212	0.49%
Collie (Smooth)	40	0.09%
Coton De Tulear	51	0.12%
Curly Coated Retriever	43	0.10%
Dachshund (Long-Haired)	39	0.09%
Dachshund (Mini Long-Haired)	168	0.39%
Dachshund (Mini Smooth-Haired)	296	0.69%
Dachshund (Mini Wire-Haired)	146	0.34%
Dachshund (Smooth-Haired)	43	0.10%
Dachshund (Wire-Haired)	94	0.22%
Dalmatian	328	0.76%
Dandie Dinmont Terrier	94	0.22%
Deerhound	87	0.20%
Dobermann	333	0.77%
Dogue de Bordeaux	139	0.32%
English Setter	183	0.43%
English Springer Spaniel	2060	4.79%
English Toy Terrier (Black & Tan)	39	0.09%
Entelbucher Mountain Dog	0	0.00%
Estrela Mountain Dog	24	0.06%
Eurasier	47	0.11%
Field Spaniel	18	0.04%
Finnish Lapphund	89	0.21%
Finnish Spitz	5	0.01%
Flat Coated Retriever	672	1.56%
Fox Terrier (Smooth)	38	0.09%
Fox Terrier (Wire)	139	0.32%
Foxhound	0	0.00%
French Bulldog	330	0.77%
German Longhaired Pointer	6	0.01%
German Pinscher	4	0.01%
German Shepherd Dog	1410	3.28%
German Shorthaired Pointer	362	0.84%
German Spitz (Klein)	23	0.05%
German Spitz (Mittel)	28	0.07%
German Wirehaired Pointer	94	0.22%
Giant Schnauzer	82	0.19%
Glen Of Imaal Terrier	33	0.08%
Golden Retriever	2059	4.79%
Gordon Setter	173	0.40%
Grand Bleu de Gascogne	0	0.00%
Great Dane	182	0.42%
Greater Swiss Mountain Dog	0	0.00%
Greenland Dog	12	0.03%
Greyhound	4	0.01%
Griffon Bruxellois	25	0.06%
Griffon Fauvre De Bretagne	0	0.00%
Hamiltonstovare	7	0.02%
Havanese	41	0.10%
Hovawart	18	0.04%
Hungarian Kuvasz	0	0.00%
Hungarian Puli	13	0.03%
Hungarian Pumi	0	0.00%
Hungarian Vizsla	441	1.03%
Hungarian Wirehaired Vizsla	173	0.40%
Ibizan Hound	19	0.04%
Irish Red & White Setter	73	0.17%
Irish Setter	382	0.89%
Irish Terrier	118	0.27%
Irish Water Spaniel	49	0.11%
Irish Wolfhound	74	0.17%
Italian Greyhound	25	0.06%
Italian Spinone	172	0.40%
Japanese Akita Inu	8	0.02%
Japanese Chin	25	0.06%
Japanese Shiba	52	0.12%
Japanese Spitz	30	0.07%
Keeshond	62	0.14%
Kerry Blue Terrier	50	0.12%
King Charles Spaniel	32	0.07%
Komondor	0	0.00%
Kooikerhondje	12	0.03%
Korean Jindo	0	0.00%
Korthals Griffon	0	0.00%
Labrador Retriever	6938	16.13%
Lagotto Romagnolo	8	0.02%
Lakeland Terrier	71	0.17%
Lancashire Heeler	27	0.06%
Large Munsterlander	80	0.19%
Leonberger	153	0.36%
Lhasa Apso	470	1.09%
Lowchen (Little Lion Dog)	19	0.04%
Maltese	68	0.16%
Manchester Terrier	72	0.17%
Maremma Sheepdog	11	0.03%
Mastiff	24	0.06%
Mexican Hairless (Intermediate)	0	0.00%
Mexican Hairless (Miniture)	0	0.00%
Mexican Hairless (Standard)	0	0.00%
Miniature Pinscher	40	0.09%
Miniature Schnauzer	1019	2.37%
Neapolitan Mastiff	10	0.02%
Newfoundland	189	0.44%
Norfolk Terrier	121	0.28%
Norwegian Buhund	21	0.05%
Norwegian Elkhound	37	0.09%
Norwich Terrier	42	0.10%
Nova Scotia Duck Tolling Retriever	130	0.30%
Old English Sheepdog	166	0.39%
Otterhound	25	0.06%
Papillon	92	0.21%
Parson Russell Terrier	209	0.49%
Pekingese	45	0.10%
Pharaoh Hound	20	0.05%
Picardy Sheepdog	0	0.00%
Pointer	241	0.56%
Polish Lowland Sheepdog	23	0.05%
Pomeranian	63	0.15%
Poodle (Miniature)	206	0.48%
Poodle (Standard)	253	0.59%
Poodle (Toy)	153	0.36%
Portuguese Podengo	14	0.03%
Portuguese Pointer (Imp)	0	0.00%
Portuguese Water Dog	35	0.08%
Pug	555	1.29%
Pyrenean Mastiff	0	0.00%
Pyrenean Mountain Dog	29	0.07%
Pyrenean Sheepdog	32	0.07%
Rhodesian Ridgeback	282	0.66%
Rottweiler	309	0.72%
Russian Black Terrier	16	0.04%
Saluki	49	0.11%
Samoyed	100	0.23%
Schipperke	19	0.04%
Schnauzer	89	0.21%
Scottish Terrier	195	0.45%
Sealyham Terrier	17	0.04%
Segugio Italiano	0	0.00%
Shar Pei	118	0.27%
Shetland Sheepdog	360	0.84%
Shih Tzu	351	0.82%
Siberian Husky	222	0.52%
Skye Terrier	17	0.04%
Sloughi	5	0.01%
Slovakian Rough Haired Pointer	0	0.00%
Small Munsterlander	0	0.00%
Soft-Coated Wheaten Terrier	147	0.34%
Spanish Water Dog	62	0.14%
St. Bernard	65	0.15%
Staffordshire Bull Terrier	797	1.85%
Sussex Spaniel	28	0.07%
Swedish Lapphund	0	0.00%
Swedish Vallhund	18	0.04%
Tibetan Mastiff	11	0.03%
Tibetan Spaniel	43	0.10%
Tibetan Terrier	402	0.93%
Turkish Kangal Dog	2	0.00%
Weimaraner	372	0.87%
Welsh Corgi (Cardigan)	45	0.10%
Welsh Corgi (Pembroke)	96	0.22%
Welsh Springer Spaniel	180	0.42%
Welsh Terrier	135	0.31%
West Highland White Terrier	835	1.94%
Whippet	707	1.64%
Yorkshire Terrier	197	0.46%
TOTAL	43,005	

There were 27,035 unique disease/condition incidents reported across 752 distinct disease/condition terms among the 43,005 dogs (see Additional file 3 for a full list). Overall, 27,972 dogs (65.04%) had no diseases/conditions reported and 15,033 (34.96%) dogs had at least one disease/condition reported.

Overall, 23,652 (87.49%) disease/condition incidents were diagnosed by a veterinary surgeon. The most commonly affected body system category was skin/ear/coat (*n* = 9786; 36.20% of all reported disorders), and the least common (other than ‘uncategorised’) was liver disorders (*n* = 277; 1.02%; Table 2). There were 101 ‘common disorders’ that had ≥50 reports in the overall survey. The individual common disorders with the highest reported overall prevalence were lipoma (*n* = 1859 reported incidents, overall prevalence of 4.32%), skin (cutaneous) cyst (*n* = 1332, 3.10%) and hypersensitivity (allergic) skin disorder (*n* = 1146, 2.66%) (Table 3).

**Table 2:** Number and proportion of disorders reported by body system category

Body system	n reports	Percent
Skin/Ear/Coat	9786	36.20%
Muscle/Bones/Joint	4561	16.87%
Digestive	2735	10.12%
Eyes	2322	8.59%
Reproductive	1462	5.41%
Nervous	1136	4.20%
Urinary	1010	3.74%
Hearts	942	3.48%
Respiratory	744	2.75%
Lump	470	1.74%
Hormonal	463	1.71%
Autoimmune	451	1.67%
Oral	359	1.33%
Blood	285	1.05%
Liver	277	1.02%
Other	32	0.12%
Total	27,035	100.00%

**Table 3:** List of number of reports and overall prevalence of the most commonly reported conditions/diseases (with ≥50 reports in unique live dogs, *n* = 43,005)

Disorder/condition	N reports	Prevalence (%)	95% CI
Acute moist dermatitis	155	0.36	(0.31–0.42)
Addison’s disease (primary hypoadrenocorticism)	56	0.13	(0.10–0.17)
Alopecia/Baldness	159	0.37	(0.32–0.43)
Anal gland/sac impaction/blockage	221	0.51	(0.45–0.59)
Anal gland/sac infection	116	0.27	(0.22–0.32)
Anal lump	74	0.17	(0.14–0.22)
Arthritis	959	2.23	(2.09–2.37)
Aural (ear) haematoma	90	0.21	(0.17–0.26)
Blindness	83	0.19	(0.16–0.24)
Blocked tear ducts	155	0.36	(0.31–0.42)
Blood present in urine	103	0.24	(0.20–0.29)
Bone cancer/tumour	88	0.2	(0.17–0.25)
Brachycephalic airway obstruction syndrome (BAOS)	50	0.12	(0.09–0.15)
Cardiomyopathy (DCM)	55	0.13	(0.10–0.17)
Cataract - age related	137	0.32	(0.27–0.38)
Chronic (long-term) kidney failure	53	0.12	(0.09–0.16)
Chronic Itching	610	1.42	(1.31–1.53)
Colitis	362	0.84	(0.76–0.93)
Conjunctivitis	260	0.6	(0.54–0.68)
Corneal Ulcer	146	0.34	(0.29–0.40)
Cruciate disease	401	0.93	(0.85–1.03)
Cruciate ligament injury	100	0.23	(0.19–0.28)
Cryptorchidism	303	0.7	(0.63–0.79)
Deafness - complete	54	0.13	(0.10–0.16)
Deafness - partial	56	0.13	(0.10–0.17)
Degenerative joint disease (DJD) (Osteoarthritis)	118	0.27	(0.23–0.33)
Demodex infestation	68	0.16	(0.12–0.20)
Dermatitis	468	1.09	(0.99–1.19)
Diabetes mellitus (sugar diabetes)	64	0.15	(0.12–0.19)
Distichiasis	137	0.32	(0.27–0.38)
Ear lump	81	0.19	(0.15–0.23)
Ear mite infestation	214	0.5	(0.44–0.57)
Elbow dysplasia	439	1.02	(0.93–1.12)
Enlarged heart	66	0.15	(0.12–0.20)
Entropion	263	0.61	(0.54–0.69)
Epilepsy	436	1.01	(0.92–1.11)
Epulis	70	0.16	(0.13–0.21)
Flea allergic dermatitis	81	0.19	(0.15–0.23)
Food Allergy	370	0.86	(0.78–0.95)
Foreign body ingestion	82	0.19	(0.15–0.24)
Gastric dilation-volvulus syndrome (GDV) / Bloat	131	0.3	(0.26–0.36)
Glaucoma	52	0.12	(0.09–0.16)
Haemorrhagic gastroenteritis (HGE) (bloody diarrhoea and vomiting)	112	0.26	(0.22–0.31)
Harvest mite (chigger) dermatitis	69	0.16	(0.13–0.20)
Heart (cardiac) failure	68	0.16	(0.12–0.20)
Heart (cardiac) murmur	384	0.89	(0.81–0.99)
Hip dysplasia	561	1.3	(1.20–1.42)
Hyperadrenocorticism (Cushing’s disease)	65	0.15	(0.12–0.19)
Hypersensitivity (allergic) skin disorder	1146	2.66	(2.52–2.82)
Hypothyroidism/Under-active thyroid	227	0.53	(0.46–0.60)
Immune Mediated Heamolytic Anaemia (IMHA)	50	0.12	(0.09–0.15)
Inflammatory bowel disease (IBD)	149	0.35	(0.30–0.41)
Intervertebral disc disorder	203	0.47	(0.41–0.54)
Irregular Heart beat	109	0.25	(0.21–0.31)
Juvenile cataract	71	0.17	(0.13–0.21)
Kennel Cough	113	0.26	(0.22–0.32)
Keratoconjunctivitis sicca (KCS Dry Eye)	236	0.55	(0.48–0.62)
Lipoma	1859	4.32	(4.13–4.52)
Lymphoma	101	0.23	(0.19–0.29)
Mammary cancer/tumour	126	0.29	(0.25–0.35)
Mammary lump	237	0.55	(0.49–0.63)
Mitral valve disease (MVD)	118	0.27	(0.23–0.33)
Nail/Claw injury	59	0.14	(0.11–0.18)
Ocular (eye) discharge	134	0.31	(0.26–0.37)
Oral (mouth) cancer/tumour	64	0.15	(0.12–0.19)
Oral (mouth) lump	72	0.17	(0.13–0.21)
Osteochondritis dissecans (OCD)	95	0.22	(0.18–0.27)
Otitis externa	629	1.46	(1.35–1.58)
Otitis media	515	1.2	(1.10–1.30)
Pancreatitis	290	0.67	(0.60–0.76)
Patellar luxation/Slipping kneecap	312	0.73	(0.65–0.81)
Persistent diarrhoea	210	0.49	(0.43–0.56)
Persistent vomiting	69	0.16	(0.13–0.20)
Persistent vomiting & diahorrea	103	0.24	(0.20–0.29)
Progressive Retinal Atrophy (PRA)	53	0.12	(0.09–0.16)
Prolapsed third eyelid gland (Cherry eye)	149	0.35	(0.30–0.41)
Pseudopregnancy (phantom pregnancy)	135	0.31	(0.27–0.37)
Pyoderma	287	0.67	(0.59–0.75)
Pyometra	244	0.57	(0.50–0.64)
Rash between folds of skin	59	0.14	(0.11–0.18)
Regular Reverse Sneezing	65	0.15	(0.12–0.19)
Seizure/Fitting	301	0.7	(0.63–0.78)
Skin (cutaneous) cyst	1332	3.1	(2.94–3.27)
Skin cancer/tumour	501	1.16	(1.07–1.27)
Skin lump	552	1.28	(1.18–1.39)
Splenic cancer/tumour	55	0.13	(0.10–0.17)
Spondylosis	112	0.26	(0.22–0.31)
Steroid Responsive Meningitis/Arteritis	63	0.15	(0.11–0.19)
Syringomyelia	74	0.17	(0.14–0.22)
Trachea/Windpipe disorder	54	0.13	(0.10–0.16)
Umbilical hernia	495	1.15	(1.05–1.26)
Unknown	127	0.3	(0.25–0.35)
Unspecified Autoimmune	144	0.33	(0.28–0.39)
Unspecified Eye	124	0.29	(0.24–0.34)
Unspecified Muscle Bone or Joint	194	0.45	(0.39–0.52)
Unspecified Respiratory	62	0.14	(0.11–0.18)
Unspecified Skin Ear or Coat	388	0.9	(0.82–1.00)
Unspecified tumour/cancer	440	1.02	(0.93–1.12)
Urinary incontinence	295	0.69	(0.61–0.77)
Urinary tract infection (UTI)	288	0.67	(0.60–0.75)
Urolithiasis (Urate crystals)	107	0.25	(0.21–0.30)

### Within breed prevalence of disorder

**Fig. 1:**
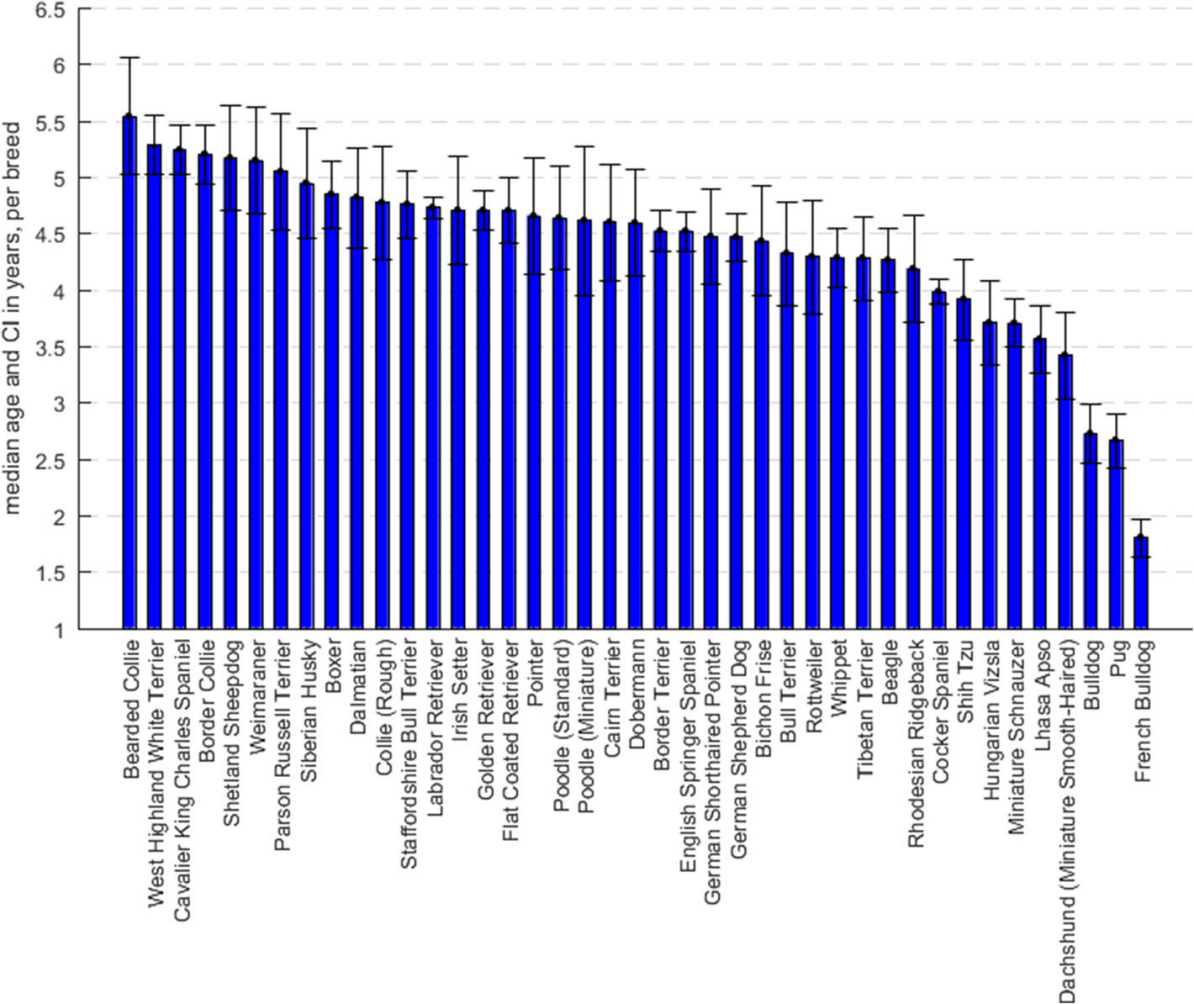
Median age (and 95% confidence intervals) of the *n* = 41 breeds with responses on ≥200 unique dogs

There were 41 ‘common breeds’ with responses on ≥200 unique live dogs per breed. These common breeds included 33,673 of the 43,005 study dogs (78.30%). The median age at the time of survey in these 41 common breeds ranged from 1.81 years for the French Bulldog to 5.54 years for the Bearded Collie (Fig. 1).

The count and proportion of dogs reported as affected by one or more diseases/conditions for each common breed are shown in Table 4. The proportion of dogs reported as unaffected by any diseases/conditions across the 41 common breeds ranged from 45.99% (Boxer) to 79.21% (Whippet). By comparison, the proportion reported as unaffected by any diseases/conditions across the entire survey [*n* = 43,005] was 65.04%.

**Table 4:** The number of unique live dogs surveyed, number of dogs with one or more reported incident of disease/condition, and clear (no reported disease/condition), affected (≥1 reported disease/condition) proportion and median age (years), for breeds with reports on ≥200 unique live dogs (*n* = 41 breeds)

Breed	n dogs	n dogs with ≥1 condition	Clear prop	Affected prop	Median age (years)
Labrador Retriever	6938	2388	65.58%	34.42%	4.73
Cocker Spaniel	3723	977	73.76%	26.24%	3.99
English Springer Spaniel	2060	670	67.48%	32.52%	4.52
Golden Retriever	2059	736	64.25%	35.75%	4.71
Border Terrier	1689	491	70.93%	29.07%	4.53
German Shepherd Dog	1410	540	61.70%	38.30%	4.47
Cavalier King Charles Spaniel	1244	607	51.21%	48.79%	5.25
Miniature Schnauzer	1019	318	68.79%	31.21%	3.71
Border Collie	1005	256	74.53%	25.47%	5.21
West Highland White Terrier	835	326	60.96%	39.04%	5.29
Staffordshire Bull Terrier	797	295	62.99%	37.01%	4.76
Boxer	724	391	45.99%	54.01%	4.85
Whippet	707	147	79.21%	20.79%	4.29
Flat Coated Retriever	672	300	55.36%	44.64%	4.71
Pug	555	216	61.08%	38.92%	2.67
Beagle	504	183	63.69%	36.31%	4.27
Lhasa Apso	470	121	74.26%	25.74%	3.57
Hungarian Vizsla	441	142	67.80%	32.20%	3.71
Tibetan Terrier	402	146	63.68%	36.32%	4.28
Irish Setter	382	160	58.12%	41.88%	4.71
Weimaraner	372	163	56.18%	43.82%	5.15
Bulldog	370	178	51.89%	48.11%	2.73
German Shorthaired Pointer	362	122	66.30%	33.70%	4.48
Shetland Sheepdog	360	126	65.00%	35.00%	5.17
Shih Tzu	351	139	60.40%	39.60%	3.92
Dobermann	333	145	56.46%	43.54%	4.60
French Bulldog	330	122	63.03%	36.97%	1.81
Dalmatian	328	137	58.23%	41.77%	4.82
Rottweiler	309	119	61.49%	38.51%	4.30
Cairn Terrier	299	102	65.89%	34.11%	4.60
Dachshund (Miniature Smooth-Haired)	296	98	66.89%	33.11%	3.42
Rhodesian Ridgeback	282	133	52.84%	47.16%	4.19
Bichon Frise	263	94	64.26%	35.74%	4.44
Poodle (Standard)	253	90	64.43%	35.57%	4.64
Pointer	241	84	65.15%	34.85%	4.66
Bearded Collie	226	80	64.60%	35.40%	5.54
Siberian Husky	222	66	70.27%	29.73%	4.95
Bull Terrier	213	110	48.36%	51.64%	4.33
Collie (Rough)	212	63	70.28%	29.72%	4.78
Parson Russell Terrier	209	61	70.81%	29.19%	5.05
Poodle (Miniature)	206	75	63.59%	36.41%	4.62

The within breed prevalence of the 101 common disorders among the 41 common breeds in the survey can be compared (Additional file 4). For the most commonly reported condition, lipoma, the within breed prevalence ranged from zero (French Bulldog) to 17.47% (Weimaraner), compared to the overall prevalence of 4.32% from all responses (*n* = 43,005) across all breeds (*n* = 187).

**Fig. 2:**
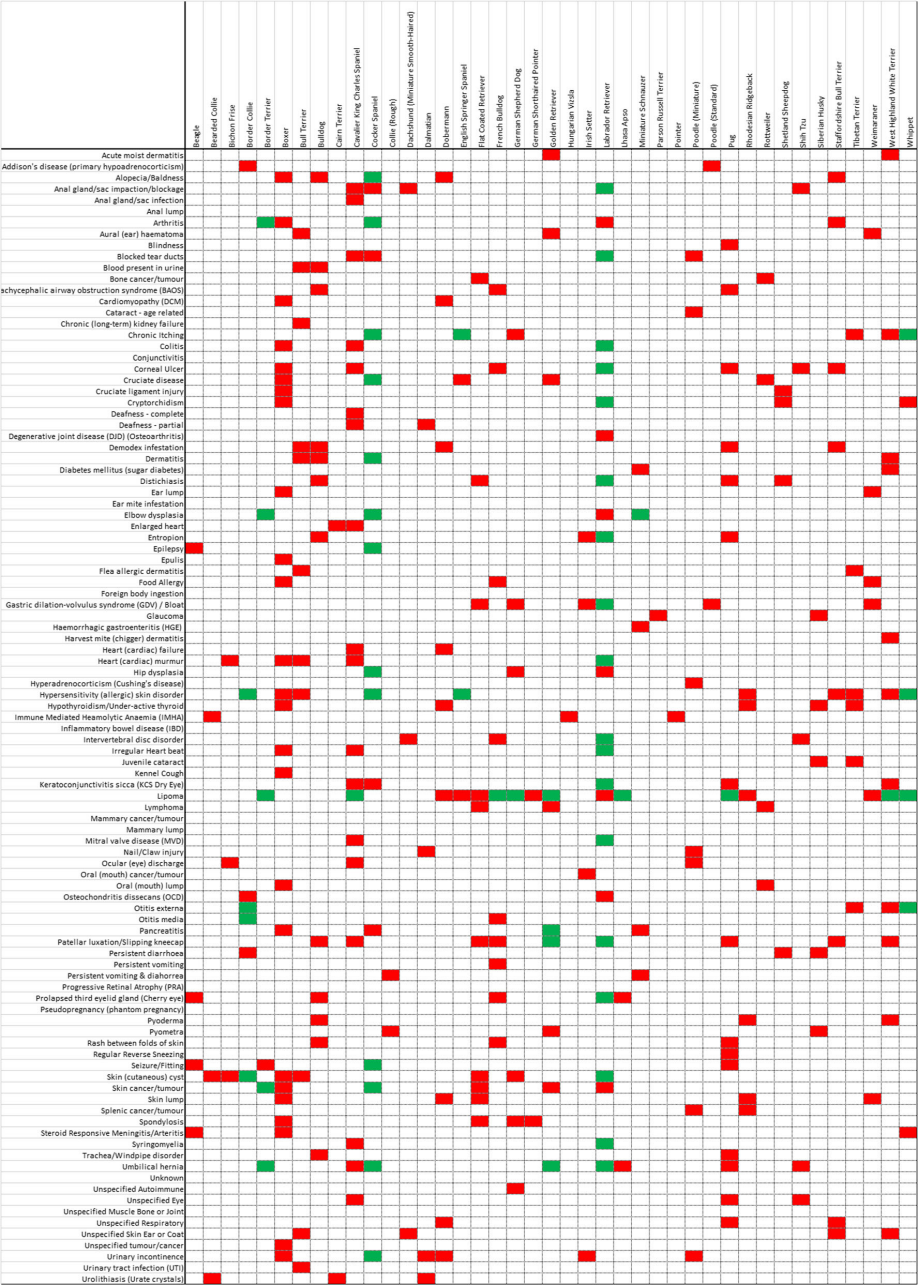
Matrix showing significantly higher (*red*) or lower (*green*) within breed prevalence than the across breeds mean prevalence of the *n* = 101 most commonly reported conditions (≥50 reports, rows) across the *n* = 41 breeds with the most responses on unique live dogs (≥200 dogs, columns). A full size version of this figure, as well as a black and white version is available in Additional files 5 and 6

Figure 2 illustrates which of the 41 common breeds has a significantly (*P* < 0.05) higher (red) and lower (green) within breed prevalence per disease/condition than the overall prevalence determined from all responses (*n* = 43,005). Ninety of the 101 (89.1%) common disorders showed a significant difference between the within breed and the overall prevalence for at least one of the 41 breeds. There was no significant difference shown in 11 diseases/conditions (anal lump, conjunctivitis, ear mite infestation, foreign body ingestion, inflammatory bowel disease, mammary cancer/tumour, mammary lump, progressive retinal atrophy, pseudopregnancy [phantom pregnancy], unknown, unspecified Muscle Bone or Joint). The disease/condition showing the greatest number of significant differences between within breed prevalence and the overall prevalence estimate was lipoma (seven breeds with a significantly higher prevalence, and nine breeds with a significantly lower prevalence).

Figure 2 also identifies within breeds which diseases/conditions have a significantly higher and lower prevalence than the overall prevalence. Using the Labrador Retriever as an example, it can be seen that of the 101 listed disorders, there are eighteen for which the Labrador Retriever has a significantly lower prevalence within breed than overall and seven diseases/conditions estimated at a significantly higher prevalence within breed than overall.

The breed with the greatest number of diseases/conditions at a significantly higher prevalence within breed than overall was the Boxer (25 disorders), while the Labrador exhibited the largest number of diseases/conditions at a significantly lower within breed prevalence than overall (18 disorders).

## Discussion

The data gathered in this survey enabled prevalence estimation and comparison for diseases/conditions both overall and by breed among a large cohort of pedigree dogs registered with the Kennel Club. The large size of the study allowed precise prevalence estimation even for relatively uncommon disorders and facilitated identification of diseases/conditions occurring at significantly higher or lower prevalence within specific breeds than determined over the whole survey (where sufficient data is available). The availability of such large volumes of data collected in a systematic format across many different breeds and disorders contributes substantially to the evidence base on health in pedigree dogs in the UK and will assist with prioritisation of the most pressing welfare concerns in many pedigree dog breeds.

The survey collected morbidity data as reported by owners on a total of 43,005 live dogs registered with the Kennel Club, making this one of the largest surveys ever of its kind. Just under two thirds of live dogs had no reported disorders. The most prevalent diseases/conditions reported across all live dogs were lipoma (4.3%), skin (cutaneous) cyst (3.1%) and hypersensitivity (allergic) skin disorder (2.7%). The most commonly affected body systems were skin/ear/coat (9786 reports), muscles/bones/joints (4561 reports) and digestive (2735 reports).

This study gathered data via an owner survey and attempted to improve the generalisability of the results by allowing inclusion of disorders that were deemed by by owners as being serious and/or persistent on up to five of their living dogs registered with the Kennel Club. The study specifically aimed also to collect data on dogs with no reported diseases/conditions in order to estimate accurately the prevalence of particular diseases/conditions in the wider population of both healthy and ill dogs. Study designs based on veterinary clinical data, although useful, may have biases towards animals with diseases/conditions making them less representative of the wider population, whilst studies using pet insurance data may additionally contain biases based on owner demographics; for example, older animals often being uninsured and financial excesses to overcome [24, 25]. However, the data collected in the current study will also have some intrinsic biases. We determined the prevalence from data as reported by participating owners and are therefore reliant on the recall, judgement on what constitutes a disorder, conscientiousness and ethics of participants, as well as they study’s ability to elicit participation. Respondents providing information on previous experiences may be affected by recall bias, where more distant events are increasingly less likely to be recalled [40, 41] which may bias against diseases/conditions that affect younger dogs, such as panosteitis. Furthermore, not all participating owners may have owned their dog from puppyhood, further exacerbating the bias against ‘early age’ reporting. However, since this is a cross-sectional study rather than a lifelong or longevity study and given the median age of 4.5 years (IQR 2.3 – 7.2 years) of dogs herein, there will also likely be a bias towards reporting of diseases/conditions tending to affect younger dogs, since there is an imbalance of data in favour early vs mature life (75% of dogs in the survey had lived longer than 2.3 years, but only 25% had lived longer than 7.2 years). This may mean diseases/conditions associated with ‘old age’, such as arthritis and laryngeal paralysis, were less likely to have been observed so far in the dogs’ lives. Age-related biases are further complicated by breed variation in longevity associated with body size and other factors [9]. Additionally, the specific wording in the survey (“…ever suffered from a serious or persistent…condition?”) means owners effectively determined the threshold for reporting a disease/condition. While this likely results in a lack of consistency in reporting mild or benign disorders, it does have an advantage of reflecting the collection of diseases/conditions that owners view as having a high welfare impact on their dogs. Therefore, despite and because of the shortcomings of the various methods of epidemiological data collection across study designs, data gathered from multiple different sources can contribute towards building more accurate pictures of the prevalence and prioritisation of diseases/conditions affecting the wider dog population.

The proportion of dogs overall reported as having experienced at least one disease/condition in this study was 35% (at a median age of 4.5 years). This leads to the question whether this indicates that the UK purebred dog population is subject to a high burden of disease? Other published data for general prevalence of affectation by disorders in dogs is limited. O’Neill et al. [6] reported that 75.8% of dogs randomly sampled from veterinary practice data were reported to be affected by at least one condition (median age = 4.8 years). Differing study methods between the current study and those of O’Neill et al. [6, 42] may account for some of this variation. Animals under veterinary care may be biased towards animals with illness but may also benefit from improved data validity by avoiding recall and misclassification bias because data were recorded contemporaneously at the time of each event and by a veterinary surgeon [22]. Furthermore, data from veterinary practices records all disorders detected, regardless of the threshold of ‘severity or persistence’ as stipulated in this survey, also possibly contributing to the higher prevalence of diseases/conditions than reported in this study.

In comparison to other species, a study on cats by O’Neill et al. [42] found 68.3% of cats had at least one disorder recorded. An Australian study estimating the prevalence of chronic diseases in human patients attending medical general practice found that 39.6% of respondents had no listed conditions diagnosed [43]. When these figures were adjusted to take into account people who did not visit a general practice doctor (so who, it may be assumed, are on the whole generally healthy and unaffected by any disorders) the estimated prevalence of being unaffected by any disease/condition was 53.2%. Purebred dog populations are subject to similar population structures and selection intensities as other domesticated species, which are very different to those in human populations, and are posited to increase the risk of disorders arising due to a high rate of inbreeding [5]. However, as companion animals, dogs are also recognised as sharing many environmental risk features with human populations which may lead to similarities in disease profiles (for example levels of diabetes mellitus and obesity are increasing in both human and dog populations in developed countries [44–46]), and dogs in the UK are often availed a more similar standard of veterinary/medical care to humans than domesticated livestock species.

Although the figures of 35% and 76% of dogs affected by at least one disease/condition reported here and by O’Neill et al. [6] may on first inspection appear rather high, they encompass a wide range of diseases/conditions affecting dogs with large variation in the severity of welfare impact on the individual. For example, this survey recorded reports that ranged from relatively minor and treatable conditions, such as skin cysts and flea allergic dermatitis, to the severe and life threatening, such as cancer and gastric dilatation volvulus/bloat. Examination of the prevalence of specific diseases/conditions is necessary to contribute towards determining the extent of the welfare burden of each across and within breeds [21].

The current study identified lipoma (4.3%), skin cyst (3.1%) and hypersensitivity (allergic) skin disorder (2.7%) as the most common disorders. A VetCompass study across the general population of dogs under primary veterinary care in the UK determined otitis externa (10.2%), periodontal disease (9.3%), anal sac impaction (7.1%) and overgrown nails (7.1%) to be the most prevalent conditions, with lipoma (3.5%) at marginally lower prevalence than, and skin masses (2.8%) and skin hypersensitivity (2.9%) at a similar prevalence to, those determined in the current study [6]. This indicates that there may indeed have been a general under-reporting of diseases/conditions considered ‘minor’ (and so below the threshold stipulated in the survey), such as some dental, anal sac and ear problems. However, prevalence estimates from this study of more serious disorders, for example those requiring minor surgery, shows concordance with estimates form other studies. A study on Danish dogs reported conditions by body system, identifying disorders of the skin as the most frequent (13.6%), which is comparable to an estimate derived from the proportion of disorders being of the skin/ear/coat and prevalence of generally affected dogs reported here (0.362 × 0.35 = 0.127). However, the second and third most frequently reported body system diseases/conditions reported in Danish dogs differed from those observed in this study (eye diseases, 13.2%; accidents, 12.6%; and diseases of the ear, 12.6%; [47]). The VetCompass study classifying conditions by organ system showed greater comparability to figures reported here, with integument (36.3%), digestive (29.5%) and musculoskeletal (14.8%) being the most frequently reported [6].

Both the current study and the VetCompass results [6] demonstrate variation in the prevalence for particular conditions across some breeds. Overall, in the current study Boxers had the greatest number (*n* = 25) of diseases/conditions with significantly higher within breed prevalence compared with overall prevalence. Four breeds had the lowest number (one) of diseases/conditions at a higher prevalence within breed than overall (Border Terrier, Hungarian Vizsla, Parson Russell Terrier and Pointer). Labradors had the greatest number of diseases/conditions at a significantly lower prevalence than overall (*n* = 18, see Fig. 2).

The particular diseases/conditions which occur at a higher prevalence in specific breeds may suggest clues underlying pathophysiological aetiology. For example, this study identifies that West Highland White Terriers appear predisposed to acute moist dermatitis, chronic itching, dermatitis, diabetes mellitus, harvest mite dermatitis, hypersensitivity (allergic) skin disorder, keratoconjunctivitis sicca, otitis externa and pyoderma, suggesting possible underlying breed weakness in the immune system [48]. Labrador Retrievers have a higher prevalence of arthritis, degenerative joint disease, elbow dysplasia, hip dysplasia, and osteochondritis dissecans suggesting that joint conditions remain a priority concern in this breed, despite decades of high participation rates in the British Veterinary Association (BVA)/Kennel Club (KC) hip dysplasia screening scheme [49] and evidence of an improving genetic trend in hip score [50]. Flat Coated Retrievers had a higher within breed prevalence of bone tumours (1.34%), lymphoma (0.89%), skin cancer (4.76%) and skin lumps (3.57%), suggesting that neoplasia may be a particular concern for this breed. This finding concurs with results from a Swedish insurance study [51]. A higher within breed than overall prevalence of a number of heart diseases/conditions in the Cavalier King Charles Spaniel were reported: enlarged heart, heart failure, heart murmur, irregular heart beat and mitral valve disease. Heart murmurs were particularly prevalent, with a within breed prevalence of 9.65% (compared to the overall prevalence of 0.89%). This finding concurs with previous reports showing that Cavalier King Charles Spaniels have a higher prevalence of cardiac conditions [16, 18, 52, 53].

Large population-based studies such as the current survey also offer opportunities to identify novel breed predispositions for diseases/conditions that have not been previously reported. For example, the current study reports a significantly higher prevalence of diabetes mellitus in West Highland White Terriers (0.96% vs. an overall prevalence of 0.16%), of entropion in Irish Setters (3.40% vs 0.61%), and spondylosis in German Shorthaired Pointers (1.38% vs 0.26%). While these figures may not appear very large (and must be interpreted with caution given potentially small numbers and non-random sampling), their availability may be valuable in raising awareness in susceptible breeds, perhaps leading to increased vigilance among owners, breeders and veterinarians. In the modern world of large datasets and so called ‘Big Data’, application of data-mining approaches offer real opportunities for hypothesis generation that can unearth novel and important findings that current hypothesis-driven methodologies are unable to address [54, 55].

The issue of under-powering because of insufficient data to support statistically significant inferences on breed predispositions is demonstrated in the results obtained in the current study for Brachycephalic Obstructive Airway Syndrome (BOAS). As expected Bulldogs, French Bulldogs and Pugs had a higher prevalence of BOAS, as has previously been reported [56]. However, Fasenella et al. [56] found BOAS to be more prevalent in the Bulldog and Pug, but also the Boston Terrier. In this study, responses were received for only 130 unique Boston Terriers and consequently they were not included in the 41 common breeds across which the differential prevalence was statistically reported. However, an inability to report robust statistical values due to paucity of data should not be interpreted as evidence that these unexplored breeds are not a breed prone to specific disorders [57].

Questions on breed health should not be limited to just identifying over-represented breeds but should also aim to identify breeds with significantly lower prevalence for certain disorders. For example, the Border Collie had a lower within breed prevalence for hypersensitivity (allergic) skin disorder, otitis externa, otitis media and skin (cutaneous) cyst, findings consistent with a Danish morbidity study [47]. Figure 2, showing significantly higher and lower within breed prevalence of diseases/conditions than the overall prevalence, allows identification of breeds which are generally less prone to disorders. For example, the Cocker Spaniel, currently the second most popular breed as judged by Kennel Club registrations, while having four diseases/conditions occurring at a higher prevalence (anal gland impaction, blocked tear ducts, keratoconjunctivitis sicca and pancreatitis), has a within breed prevalence lower than the overall prevalence for 13 disorders: alopecia/baldness, arthritis, chronic itching, cruciate disease, dermatitis, elbow dysplasia, epilepsy, hip dysplasia, hypersensitivity (allergic) skin disorder, seizure, skin tumours, umbilical hernia and urinary incontinence. The Border Terrier is more prone to just one disorder (seizures), while being less prone to five conditions: arthritis, elbow dysplasia, lipoma, skin tumours and umbilical hernia. This highlights the complexity that exists for each breed as it faces its own particular concerns and while some breeds are prone to certain conditions, they may be less so to others. The results of this study will contribute to the limited information currently available and it is anticipated that it will be used in the forthcoming Breed Health and Conservation Plans to assist in the identification and prioritisation of selection objectives and in providing advice to breeders/owners on common conditions in particular breeds that may assist in minimising risk. However, future breed-specific studies are recommended to report more precise prevalence estimates across more breeds than the current study could include.

It should be noted, however, that the variation in median and IQR of age at the time of the survey in these 41 common breeds (shown in Fig. 1), implies that these disorder results may be subject to age-related biases as discussed earlier. In particular, the young median age of French Bulldogs, Pugs and Bulldogs in this study (Fig. 1) may have hampered the ability to accurately determine the prevalence of diseases/conditions tending to affect dogs later in life (for example, arthritis and age-related cataracts). Conversely, the higher median age of Cavalier King Charles Spaniels in this study may account in part for the relatively high prevalence of diseases/conditions as the dogs in the study would have each contributed greater time to the study during which to have disorder events. In general, across all breeds, the imbalance of ‘early’ vs ‘later life’ data would be expected to bias against the detection of diseases/conditions primarily affecting older dogs. However, this survey does provide useful data on the disorders and conditions currently affecting pedigree breeds in what may be regarded as their ‘prime of life’.

There is likely to be considerable variation in the welfare impact across the range of diseases/conditions included in this study, which vary from acute to chronic, treatable to incurable, and life-threatening to merely irritating. However, every disease/condition reported will have resulted in some impact on the dog’s welfare and a financial and/or emotional cost to the owner, and is therefore important. It is hoped that the results presented here will assist a wide range of stakeholders involved in the prevention, treatment and management of disorders in dogs. For example, the within breed prevalence of conditions such as food allergies and urinary incontinence may be primarily of interest to owners, more acute and severe conditions like gastric dilatation volvulus (bloat) and epilepsy may be of more interest to veterinary professionals who treat dogs and advise owners, whilst some conditions which have an equivalent in human medicine or where breed-specific conditions are little known may be of more interest to translational researchers [58]. Data such as those presented in the current study are critical to optimise the allocation of resources for the greatest impact in alleviating suffering and improving welfare, thereby contributing to improving disease control through prioritisation, decision making and animal husbandry [6, 59, 60]. Some diseases/conditions which may be considered as clinically benign may actually have a large overall welfare impact due to high prevalence or duration within or across breeds. Thus techniques such as the generic illness severity index for dogs (GISID), a scale proposed by Asher et al. [2], which aims to categorise and prioritise the overall welfare impact of disorders based on severity and prevalence, may assist in determining the total welfare impact of specific conditions.

There is substantial evidence of welfare improvements from official health schemes, amending breed standards, and developing estimated breeding values for complex inherited conditions that the current study aimed to optimise [61–66]. However, the current results also clearly indicate the need for ongoing participation in existing screening schemes, and the benefits from development of new methods to gather widespread data on prevalence, duration and severity of diseases/conditions in order to prioritise the focused delivery of health schemes overall and within specific breeds towards those areas with the highest prospects of real welfare improvement at population levels.

## Conclusion

This report describes the most prevalent diseases/conditions among Kennel Club registered dogs overall and within common breeds. Just under two-thirds of live dogs had no reported conditions, whilst the most prevalent specific conditions reported were lipoma, skin (cutaneous) cyst and hypersensitivity (allergic) skin disorder. When conditions were classified by body system, the most prevalent body systems affected were skin/ear/coat, muscles/bones/joints and digestive. The information collected through this study has added to valuable epidemiological data and it is anticipated that it will be used to assist in the identification and prioritisation of selection objectives and in providing advice to breeders/owners on common conditions in particular breeds that may be taken to minimise risk.

## Additional files

Additional file 1: The questionnaire questions. (PDF 1124 kb)

Additional file 2: Complete list of conditions supplied for each body/system category in the survey. (XLSX 13 kb)

Additional file 3: List of all 752 diseases/conditions reported across the 43,005 unique live dogs surveyed, number of incidents reported and overall prevalence. (XLSX 41 kb)

Additional file 4: The within breed prevalence (and 95% confidence intervals) of the most commonly reported 101 conditions (with ≥50 reports in all unique live dogs) across the 41 breeds with most responses for unique live dogs (≥200 unique live dogs). (XLSX 36 kb)

Additional file 5: Full size version of Figure 2. (PNG 143 kb)

Additional file 6: Black and white version of Figure 2. (PNG 146 kb)

## Abbreviations

BOAS: Brachycephalic Obstructive Airway Syndrome
BVA: British Veterinary Association
CEVM: Centre for Evidence-Based Veterinary Medicine
CI: Confidence interval
GISID: Generic Illness Severity Index for Dogs
IQR: Inter-quartile range
KC: Kennel Club

## Footnotes

1 Refers to the breeds recognised at the time of the survey. Two breeds have been recognised by The Kennel Club since the survey closed; the Braque D’Auvergne and Jack Russell.
