# Supplementary material for: Large-scale survey to estimate the prevalence of disorders for 192 Kennel Club registered breeds
Source: Canine Genet Epidemiol. 2017 Sep 19;4:8. doi: 10.1186/s40575-017-0047-3 (PMC5604186; doi:10.1186/s40575-017-0047-3)
Supplement: Supplementary file 1 — The questionnaire questions. (PDF 1124 kb) [file 40575_2017_47_MOESM1_ESM.pdf]

This survey is being carried out for Kennel Club breed registered dogs living and those that have died since 2004.
This survey currently does not include activity and companion registered dogs.

Please fill in the survey for:

- Each dog that is **registered with the Kennel Club on the Breed register** (this does not include dogs only registered on companion or activity register)
- Each dog that is **older than six months**
- Each dog that has sadly passed away **since the 31st December 2004**

To fill in the survey we recommend you have the following to hand:

- **Kennel Club Registration documentation** – including the Kennel Club registered name and number
- Any **veterinary records** for your dog(s)
- Any **records of litters** of puppies

If at any point during the survey you want to go back to the previous page, please press the ‘previous’ button at the bottom of the page, **do not** go back on your internet browser.

***1. Do you currently own any living dog(s)?**

- Yes
- No

Section A: General Information on dogs you currently own

2. Please enter each dog's Kennel Club registered number (please take note of each dogs number to ensure the results match up throughout the survey).

Dog 1

Dog 2

Dog 3

Dog 4

Dog 5

3. Please enter each dog's Kennel Club registered name (please match this to the dog number and dogs registration number).

Dog 1

Dog 2

Dog 3

Dog 4

Dog 5

Please fill in at minimum, for each of your dogs: the Kennel Club registered name or the Kennel Club registered number. **If you do not fill in either, your survey will be void**.

If you have over 5 dogs which are over 6 months old, you should fill in the survey on your oldest, 2nd oldest, middle aged, 2nd youngest and youngest dog.

Please take note of each dog's number to ensure the results match up throughout the survey.

***4. Please select each dog's breed**

	Breeds
Dog 1	
Dog 2	
Dog 3	
Dog 4	
Dog 5	

5. Please fill in the date of birth for each dog.
(If you do not know the exact date, please complete as much as possible)

	Day	Month	Year
Dog 1			
Dog 2			
Dog 3			
Dog 4			
Dog 5			

6. Please fill in the sex of each dog and whether they are neutered?

	Sex:	Neutered:	Age neutered
Dog 1			
Dog 2			
Dog 3			
Dog 4			
Dog 5			

7. Have your dog(s) taken part in any of the following activities?:

	Agility	Field Trials/Working trials	Flyball	Heelwork to music	Obedience competitions	Rally	Showing	None
Dog 1	☐	☐	☐	☐	☐	☐	☐	☐
Dog 2	☐	☐	☐	☐	☐	☐	☐	☐
Dog 3	☐	☐	☐	☐	☐	☐	☐	☐
Dog 4	☐	☐	☐	☐	☐	☐	☐	☐
Dog 5	☐	☐	☐	☐	☐	☐	☐	☐

Other (please specify and which dog)

8. Where did you get each dog from?

Dog 1	
Dog 2	
Dog 3	
Dog 4	
Dog 5	

Other (please specify and which dog)

9. Did your vet comment on your dogs body condition or weight on your most recent visit, please fill in below:

	Body condition	Dogs age when comment was made
Dog 1		
Dog 2		
Dog 3		
Dog 4		
Dog 5		

Section B: Health conditions affecting dogs you currently own

Please note that we would like information on all conditions, not just those known to be inherited.

***10. Have any of the dogs included in this survey ever suffered from any growths, lumps or cancers?**

- Yes
- No

11. Please select the lump, growth or cancer, the age at which each dog was first affected and whether they were diagnosed by a vet.

	Condition 1	Age first affected by conditon 1	Was condition 1 diagnosed by a vet?	Condition 2	Age first affected by condition 2	Was condition 2 diagnosed by a vet?
Dog 1						
Dog 2						
Dog 3						
Dog 4						
Dog 5						

12. Other or additional cancers/tumours/lumps (please specify condition, the age at which each dog was first affected and whether they were diagnosed by a vet)

Dog 1

Dog 2

Dog 3

Dog 4

Dog 5

***13. Have any of the dogs included in this survey ever suffered from an autoimmune condition(s)?
(including immune-mediated conditions)**

- ○ Yes
- ○ No

14. Please select the name of the autoimmune condition(s), the age at which each dog was first affected and whether they were diagnosed by a vet.

	Autoimmune condition 1	Age first affected by condition 1	Was condition 1 diagnosed by a vet?	Autoimmune condition 2	Age first affected by condition 2	Was condition 2 diagnosed by a vet?
Dog 1						
Dog 2						
Dog 3						
Dog 4						
Dog 5						

15. Other or additional autoimmune condition:
(please specify the condition, the age at which each dog was first affected and whether they were diagnosed by a vet)

Dog 1	
Dog 2	
Dog 3	
Dog 4	
Dog 5	

***16. Have any of the dogs included in this survey ever suffered from a serious or persistent oral or dental condition(s)?**

- ○ Yes
- ○ No

17. Please select the name of the oral and dental condition(s), the age at which each dog was first affected and whether they were diagnosed by a vet.

	Oral/dental condition 1	Age first affected by condition 1	Was condition 1 diagnosed by a vet?	Oral/dental condition 2	Age first affected by condition 2	Was condition 2 diagnosed by a vet?
Dog 1						
Dog 2						
Dog 3						
Dog 4						
Dog 5						

18. Other or additional oral/dental condition:
(please specify the condition, the age at which each dog was first affected and whether they were diagnosed by a vet)

Dog 1	
Dog 2	
Dog 3	
Dog 4	
Dog 5	

***19. Have any of the dogs included in this survey ever suffered from a serious or persistent digestive system condition(s)?**
(including conditions affecting the oesophagus, stomach, pancreas, intestines, colon and anus, whilst excluding diabetes)

- ○ Yes
- ○ No

20. Please select the name of the digestive system condition(s), the age at which each dog was first affected and whether they were diagnosed by a vet.

	Digestive condition 1	Age first affected by condition 1	Was condition 1 diagnosed by a vet?	Digestive condition 2	Age first affected by condition 2	Was condition 2 diagnosed by a vet?
Dog 1						
Dog 2						
Dog 3						
Dog 4						
Dog 5						

21. Other or additional digestive condition: (please specify the condition, the age at which each dog was first affected and whether they were diagnosed by a vet)

Dog 1

Dog 2

Dog 3

Dog 4

Dog 5

***22. Have any of the dogs included in this survey ever suffered from a serious or persistent heart condition(s)?**

- ○ Yes
- ○ No

23. Please select the name of the heart condition(s), the age at which each dog was first affected and whether they were diagnosed by a vet.

	Heart condition 1	Age first affected by condition 1	Was condition 1 diagnosed by a vet?	Heart condition 2	Age first affected by condition 2	Was condition 2 diagnosed by a vet?
Dog 1						
Dog 2						
Dog 3						
Dog 4						
Dog 5						

24. Other or additional heart condition:
(please specify the condition, the age at which each dog was first affected and whether they were diagnosed by a vet)

Dog 1	
Dog 2	
Dog 3	
Dog 4	
Dog 5	

***25. Have any of the dogs included in this survey ever suffered from a serious or persistent respiratory (breathing) condition(s)?**
(including conditions affecting the nose, airways and lungs)

- ○ Yes
- ○ No

26. Please select the name of the respiratory system condition(s), the age at which each dog was first affected and whether they were diagnosed by a vet.

	Respiratory condition 1	Age first affected by condition 1	Was condition 1 diagnosed by a vet?	Respiratory condition 2	Age first affected by condition 2	Was condition 2 diagnosed by a vet?
Dog 1						
Dog 2						
Dog 3						
Dog 4						
Dog 5						

27. Other or additional respiratory condition:
(please specify the condition, the age at which each dog was first affected and whether they were diagnosed by a vet)

Dog 1

Dog 2

Dog 3

Dog 4

Dog 5

***28. Have any of the dogs included in this survey ever suffered from a serious or persistent eye condition(s)?**

- Yes
- No

29. Please select the name of the eye condition(s), the age at which each dog was first affected and whether they were diagnosed by a vet.

	Eye condition 1	Age first affected by condition 1	Was condition 1 diagnosed by a vet?	Eye condition 2	Age first affected by condition 2	Was condition 2 diagnosed by a vet?
Dog 1						
Dog 2						
Dog 3						
Dog 4						
Dog 5						

30. Other or additional eye condition:
(please specify the condition, the age at which each dog was first affected and whether they were diagnosed by a vet)

Dog 1

Dog 2

Dog 3

Dog 4

Dog 5

***31. Have any of the dogs included in this survey ever suffered from a serious or persistent ear, skin or coat condition(s)?**

- Yes
- No

32. Please select the name of the ear, skin or coat condition(s), the age at which each dog was first affected and whether they were diagnosed by a vet.

	Ear, skin or coat condition 1	Age first affected by condition 1	Was condition 1 diagnosed by a vet?	Ear, skin or coat condition 2	Age first affected by condition 2	Was condition 2 diagnosed by a vet?
Dog 1						
Dog 2						
Dog 3						
Dog 4						
Dog 5						

33. Other or additional ear, skin or coat condition:
(please specify the condition, the age at which each dog was first affected and whether they were diagnosed by a vet)

Dog 1

Dog 2

Dog 3

Dog 4

Dog 5

***34. Have any of the dogs included in this survey ever suffered from a serious or persistent condition(s) affecting the muscles, bones or joints?
(including hernias)**

- ○ Yes
- ○ No

35. Please select the name of the muscle, bone or joint condition(s), the age at which each dog was first affected and whether they were diagnosed by a vet.

	Muscle, bone or joint condition 1	Age first affected by condition 1	Was condition 1 diagnosed by a vet?	Muscle, bone or joint condition 2	Age first affected by condition 2	Was condition 2 diagnosed by a vet?
Dog 1						
Dog 2						
Dog 3						
Dog 4						
Dog 5						

36. Other or additional muscle, bone or joint conditions: (please specify the condition, the age at which each dog was first affected and whether they were diagnosed by a vet)

Dog 1	
Dog 2	
Dog 3	
Dog 4	
Dog 5	

***37. Have any of the dogs included in this survey ever suffered from a serious or persistent nervous system condition(s)?**
(including epilepsies and seizures)

- ○ Yes
- ○ No

38. Please select the name of the nervous system condition(s), the age at which each dog was first affected and whether they were diagnosed by a vet.

	Nervous system condition 1	Age first affected by condition 1	Was condition 1 diagnosed by a vet?	Nervous system condition 2	Age first affected by condition 2	Was condition 2 diagnosed by a vet?
Dog 1						
Dog 2						
Dog 3						
Dog 4						
Dog 5						

39. Other or additional nervous system conditions: (please specify the condition, the age at which each dog was first affected and whether they were diagnosed by a vet)

Dog 1

Dog 2

Dog 3

Dog 4

Dog 5

***40. Have any of the dogs included in this survey ever suffered from a serious or persistent reproductive condition(s)?**
(including genital and mammary conditions)

- ○ Yes
- ○ No

41. Please select the name of the reproductive system condition(s), the age at which each dog was first affected and whether they were diagnosed by a vet.

	Reproductive condition 1	Age first affected by condition 1	Was condition 1 diagnosed by a vet?	Reproductive condition 2	Age first affected by condition 2	Was condition 2 diagnosed by a vet?
Dog 1						
Dog 2						
Dog 3						
Dog 4						
Dog 5						

42. Other or additional reproductive system conditions:
(please specify the condition, the age at which each dog was first affected and whether they were diagnosed by a vet)

Dog 1

Dog 2

Dog 3

Dog 4

Dog 5

***43. Have any of the dogs included in this survey ever suffered from a serious or persistent liver condition(s)?**

- ◯ Yes
- ◯ No

44. Please select the name of the liver condition(s), the age at which each dog was first affected and whether they were diagnosed by a vet.

	Liver condition 1	Age first affected by condition 1	Was condition 1 diagnosed by a vet?	Liver condition 2	Age first affected by condition 2	Was condition 2 diagnosed by a vet?
Dog 1						
Dog 2						
Dog 3						
Dog 4						
Dog 5						

45. Other or additional liver conditions: (please specify the condition, the age at which each dog was first affected and whether they were diagnosed by a vet)

Dog 1	
Dog 2	
Dog 3	
Dog 4	
Dog 5	

***46. Have any of the dogs included in this survey ever suffered from a serious or persistent urinary tract condition(s)?**

- ○ Yes
- ○ No

47. Please select the name of the urinary tract condition(s), the age at which each dog was first affected and whether they were diagnosed by a vet.

	Urinary tract condition 1	Age first affected by condition 1	Was condition 1 diagnosed by a vet?	Urinary condition 2	Age first affected by condition 2	Was condition 2 diagnosed by a vet?
Dog 1						
Dog 2						
Dog 3						
Dog 4						
Dog 5						

48. Other or additional urinary conditions:
(please specify the condition, the age at which each dog was first affected and whether they were diagnosed by a vet)

Dog 1

Dog 2

Dog 3

Dog 4

Dog 5

***49. Have any of the dogs included in this survey ever suffered from a serious or persistent blood condition(s)?
(including conditions affecting the spleen and lymph nodes)**

- Yes
- No

50. Please select the name of the blood condition(s), the age at which each dog was first affected and were they diagnosed by a vet?

	Blood condition 1	Age first affected by condition 1	Was condition 1 diagnosed by a vet?	Blood condition 2	Age first affected by condition 2	Was condition 2 diagnosed by a vet?
Dog 1						
Dog 2						
Dog 3						
Dog 4						
Dog 5						

51. Other or additional blood conditions:
(please specify the condition, the age at which each dog was first affected and whether they were diagnosed by a vet)

Dog 1

Dog 2

Dog 3

Dog 4

Dog 5

***52. Have any of the dogs included in this survey ever suffered from a serious or persistent hormonal (endocrine) condition(s) (including diabetes)?**

- Yes
- No

53. Please select the name of the hormonal (endocrine) condition(s), the age at which each dog was first affected and whether they were diagnosed by a vet.

	Hormonal (endocrine) condition 1	Age first affected by condition 1	Was condition 1 diagnosed by a vet?	Hormonal (endocrine) condition 2	Age first affected by condition 2	Was condition 2 diagnosed by a vet?
Dog 1						
Dog 2						
Dog 3						
Dog 4						
Dog 5						

54. Other or additional hormonal conditions:
(please specify the condition, the age at which each dog was first affected and whether they were diagnosed by a vet)

Dog 1

Dog 2

Dog 3

Dog 4

Dog 5

***55. Have any of the dogs included in this survey ever suffered from any other serious or persistent condition(s) not covered in the previous questions?**

- Yes
- No

***56. Please name the other condition(s) your dog has suffered from, age first affected (years, months) and whether they were diagnosed by a vet:**

Dog 1	
Dog 2	
Dog 3	
Dog 4	
Dog 5	

Section C: Breeding a litter

This section is about dams you currently own, which are included in this survey and their litters.

*57. If any of the dogs included in the survey are female, have they bred a litter?

- ◯ Yes
- ◯ No
- ◯ None are female

58. Have any of these breeding female dog(s), been tested by any of the following, prior to first being bred?
(Please leave non-breeding females and males blank)

	BVA/KC schemes	Breed club schemes	DNA health tests
Dog 1			
Dog 2			
Dog 3			
Dog 4			
Dog 5			

59. How many litters have the breeding female dogs had? (Please leave non-breeding females and males as blank)

	Number of litters:
Dog 1	
Dog 2	
Dog 3	
Dog 4	
Dog 5	

60. What were the age(s) of these female dog(s), when they had their first season?
(Please leave non-breeding females and males blank)

	Age at first season:
Dog 1	
Dog 2	
Dog 3	
Dog 4	
Dog 5	

61. What age were the female dogs when they had each of their litters?
(Please leave non-breeding females and males blank)

	Litter 1	Litter 2	Litter 3	Litter 4	Litter 5	Litter 6
Dog 1						
Dog 2						
Dog 3						
Dog 4						
Dog 5						

62. For each female dog please specify how each litter was delivered:
(Please leave non-breeding females and males blank)

	Litter 1	Litter 2	Litter 3	Litter 4	Litter 5	Litter 6
Dog 1						
Dog 2						
Dog 3						
Dog 4						
Dog 5						

The following questions are asking about the puppies from each litter your female(s) have had.

63. How many puppies were born in total for each litter (both dead and alive)?

	Litter 1	Litter 2	Litter 3	Litter 4	Litter 5	Litter 6
Dog 1						
Dog 2						
Dog 3						
Dog 4						
Dog 5						

64. How many puppies were born alive in each litter?

	Litter 1	Litter 2	Litter 3	Litter 4	Litter 5	Litter 6
Dog 1						
Dog 2						
Dog 3						
Dog 4						
Dog 5						

65. How many puppies were alive at 8 weeks for each litter?

	Litter 1	Litter 2	Litter 3	Litter 4	Litter 5	Litter 6
Dog 1						
Dog 2						
Dog 3						
Dog 4						
Dog 5						

66. Have any of the puppies from these litters ever suffered from a birth defect or other congenital conditions?

- Yes
- No

67. Please name the condition(s) that your puppies suffered from, in line with the mother's dog number (please separate conditions with a comma)

Dog 1

Dog 2

Dog 3

Dog 4

Dog 5

Section D: Using your dog at Stud

This section is about stud dogs that you currently own, that are included in this survey.

*68. If any of the dogs used in this survey are male, have any of them been used at stud?

- Yes
- No
- None are male

69. Have any of these stud dog(s), been tested by any of the following, prior to first being bred?
(Please leave non-stud males and females blank)

	BVA/KC schemes	Breed club Schemes	DNA health tests
Dog 1			
Dog 2			
Dog 3			
Dog 4			
Dog 5			

70. Are you aware of the Kennel Club resource Mate select and have you used it?

- Yes, I am aware of Mate select and have used it
- Yes, I am aware of Mate select, but have not used it
- No, I am not aware of Mate select

71. Please rank the following in order of importance to you in relation to breeding dogs?
(Where 1 is the highest importance and 5 is the lowest importance - these will reorder themselves accordingly)

- Genetic diversity/COI
- Health test results
- Kennel Club Breed Standards
- Demand/request for puppies
- Awards (e.g. from shows or other activities)
- Temperament

Section E: Behaviour

72. Tick all behaviours that apply to your dog:

	Dog 1	Dog 2	Dog 3	Dog 4	Dog 5
Responds immediately to basic commands (Sit, stay, waits, down)	☐	☐	☐	☐	☐
Walks to heel and does not pull	☐	☐	☐	☐	☐
Responds immediately if recalled when off lead	☐	☐	☐	☐	☐
Is friendly to people	☐	☐	☐	☐	☐
Is friendly with other adult dogs	☐	☐	☐	☐	☐
Is friendly with young dogs or puppies	☐	☐	☐	☐	☐
Is playful	☐	☐	☐	☐	☐
Is easy to control	☐	☐	☐	☐	☐
Is calm and quiet	☐	☐	☐	☐	☐
May exhibit aggressive signs towards dogs (barking, growling, raised hackles, snapping)	☐	☐	☐	☐	☐
May exhibit aggressive signs towards people (barking, growling, raised hackles, snapping)	☐	☐	☐	☐	☐
Exhibits destructive behaviour (chews or scratches furniture or other household objects)	☐	☐	☐	☐	☐
Barks excessively	☐	☐	☐	☐	☐
Appears uneasy or anxious before or during travel	☐	☐	☐	☐	☐
Appears uneasy or anxious around children	☐	☐	☐	☐	☐
Sometimes shows protective behaviour or guards people, food or items	☐	☐	☐	☐	☐
Jumps up at people	☐	☐	☐	☐	☐
Is excessive, difficult to control and if it lunges is hard to hold back	☐	☐	☐	☐	☐
Chews/destroys, soils or barks/howls when you leave the house	☐	☐	☐	☐	☐
Shows a fearful response to noises or storms	☐	☐	☐	☐	☐
Is spooked by odd or unexpected things or objects	☐	☐	☐	☐	☐
Is anxious or uneasy in	☐	☐	☐	☐	☐

new situations

73. Any additional behavioural information about each dog:

Dog 1	
Dog 2	
Dog 3	
Dog 4	
Dog 5	

Section F: Cause of Death

This section is about any adult dogs that you have owned and that have died, since 2004*.

For each dog over the age of 8 weeks, that has died (or been euthanised) since 2004, please tell us about the cause of death or reason for euthanasia. Please be as specific as possible in describing the cause of death, using the diagnosis made by your veterinary surgeon whenever possible. If you have difficulty remembering, please consider contacting your veterinary practice to ask.

*Due to the results collected in the 2004 Kennel Club study.

*74. Have any of your dog(s) been euthanised or died, since 2004?

- ○ Yes
- ○ No

75. Please enter the Kennel Club registration number of the dog(s) that have been euthanised or died.

Dog A

Dog B

Dog C

Dog D

Dog E

76. Please enter the Kennel Club registered name of the dog(s) that have been euthanised or died.

Dog A

Dog B

Dog C

Dog D

Dog E

77. What was the date of birth for your dogs?
(If exact date is unknown, please fill in as much as possible)

	Day	Month	Year
Dog A			
Dog B			
Dog C			
Dog D			
Dog E			

78. How did your dog(s) die?

Dog A

Dog B

Dog C

Dog D

Dog E

79. Please provide the reason(s) for euthanasia or cause(s) of death, if known (please be as specific as possible).

Dog A

Dog B

Dog C

Dog D

Dog E

80. Was a post mortem (autopsy) was performed?

	Was a post mortem (autopsy) performed?
Dog A	
Dog B	
Dog C	
Dog D	
Dog E	

81. What was the date of your dogs death?
(If exact date is unknown, please fill in as much as possible)

	Day	Month	Year
Dog A			
Dog B			
Dog C			
Dog D			
Dog E			

82. Are you happy to share the information that your dog(s) have died with Kennel Club registration department, so that the Kennel Club will not contact you regarding the deceased dog(s)?

- Yes
- No

83. Please feel free to leave any comments you have on the survey or any other relevant information about your dog(s). Your input is welcomed.

84. Would you like to be included in the following?

☐ I would like to be contacted for future health studies.

☐ I would like to be entered for the prize draw.

Please enter your email address if you have ticked either of the above:

85. If you have provided your email address above, then the Kennel Club or our marketing partners may contact you by email to tell you about our related services and to send you details of special offers and discounts available.
By providing us with your email address and registering with us you consent to being contacted by these methods for these purposes.

☐ If you do not wish to receive special offers, discounts and information from the Kennel Club or our marketing partners please tick this box.

☐ or from carefully selected third parties tick this box.
