# Supplementary figures and images for: Large-scale survey to estimate the prevalence of disorders for 192 Kennel Club registered breeds
Source: Canine Genet Epidemiol. 2017 Sep 19;4:8. doi: 10.1186/s40575-017-0047-3 (PMC5604186; doi:10.1186/s40575-017-0047-3)

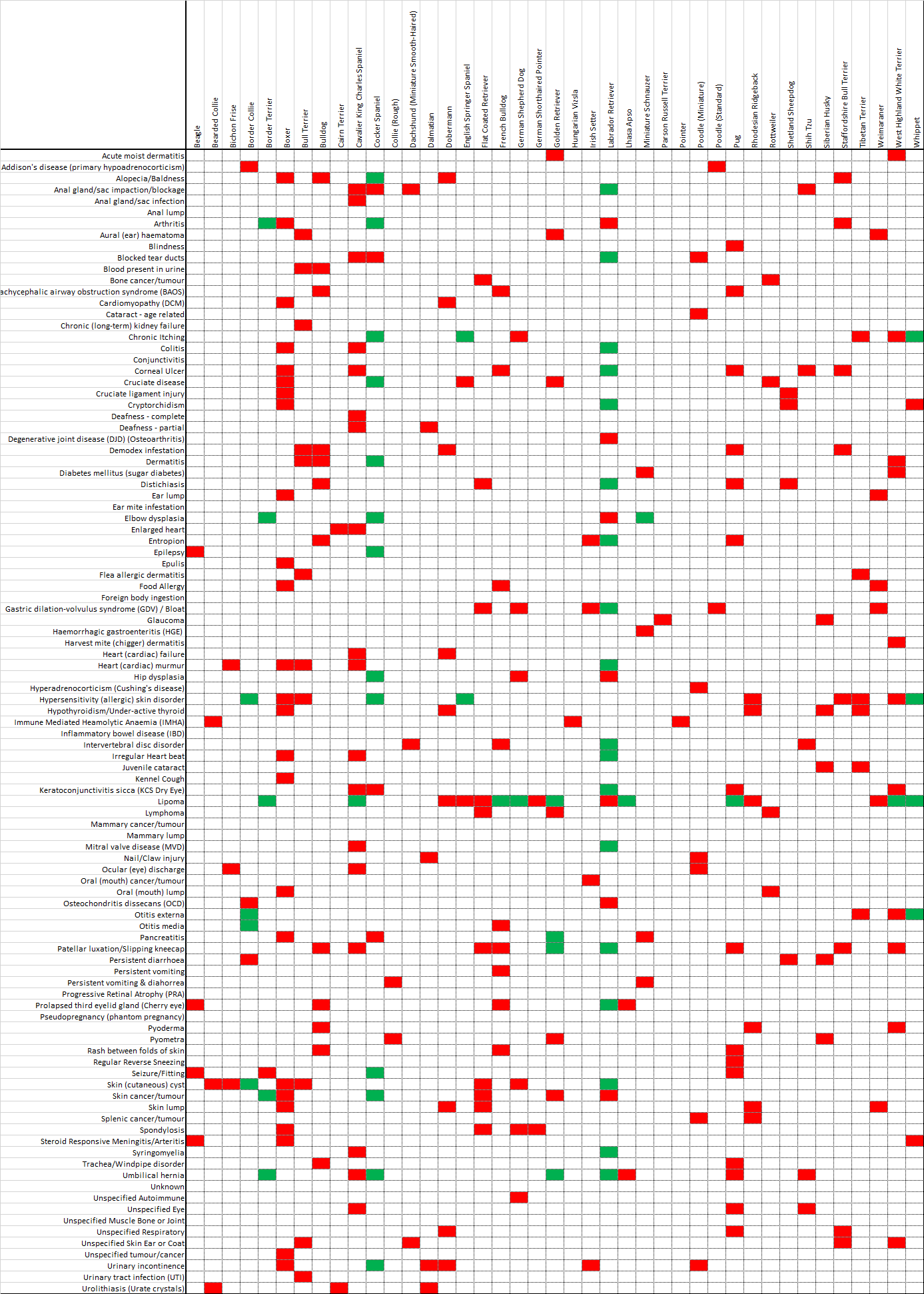

Supplement: Supplementary file 5 — Full size version of Figure 2. (PNG 143 kb) [file 40575_2017_47_MOESM5_ESM.png]

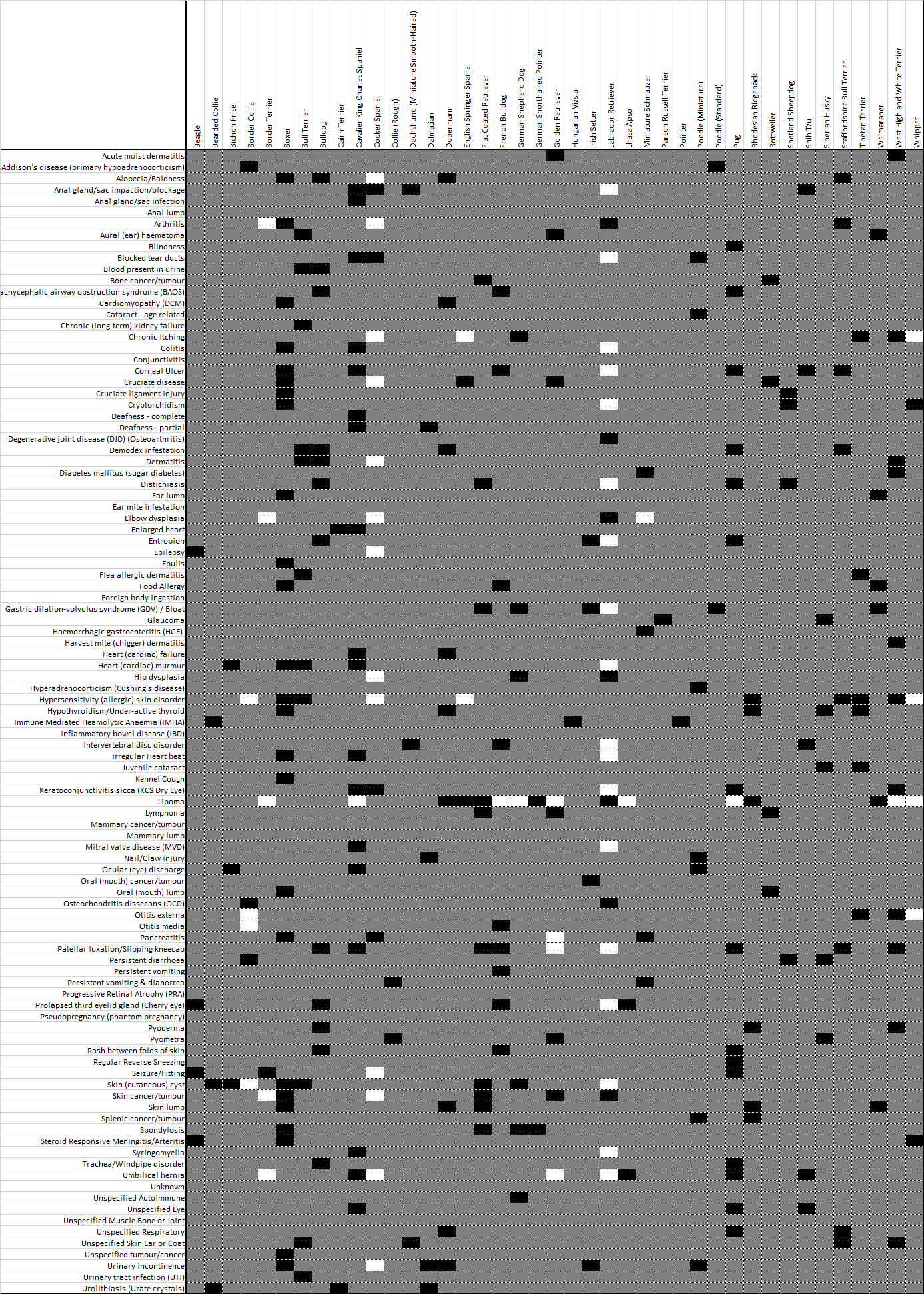

Supplement: Supplementary file 6 — Black and white version of Figure 2. (PNG 146 kb) [file 40575_2017_47_MOESM6_ESM.png]
